# Birds with multiple homes. The annual cycle of the pallid swift (*Apus pallidus brehmorum*)

**DOI:** 10.1371/journal.pone.0259656

**Published:** 2021-11-30

**Authors:** Stewart Finlayson, Tyson Lee Holmes, Geraldine Finlayson, Rhian Guillem, Charles Perez, Keith Bensusan, Clive Finlayson

**Affiliations:** 1 The Gibraltar National Museum, Gibraltar, United Kingdom; 2 Institute of Life and Earth Sciences, The University of Gibraltar, Gibraltar, United Kingdom; 3 Department of Life Sciences, Liverpool John Moores University, Liverpool, United Kingdom; 4 Gibraltar Botanic Gardens, ‘The Alameda’, Gibraltar, United Kingdom; 5 Gibraltar Ornithological and Natural History Society, Gibraltar, United Kingdom; 6 Department of Social Sciences, University of Toronto Scarborough, Toronto, Canada; MARE – Marine and Environmental Sciences Centre, PORTUGAL

## Abstract

We tracked pallid swifts (*Apus pallidus brehmorum*) from a single breeding colony in Gibraltar over two years. Our results show movement of birds between specific regions within the non-breeding geographical area at specific times of the year. The tracking of a single individual showed remarkable fidelity to the areas visited between years. Furthermore, two pallid swifts tracked over the entire eight-month non-breeding period, while in Africa, gave no indication of coming to land, supporting previous findings of an airborne existence in swifts outside the breeding season. In addition, the crossing of the Sahara Desert to and from breeding grounds is remarkably fast, with one individual crossing it in just over a day. We discuss our findings in the context of bird migration evolutionary strategies.

## Introduction

The classic view of migratory birds is one of species that move annually between two homes [1–3]. In this long-established view migratory birds migrate between breeding grounds and winter quarters, and back. This perspective of migration has been conditioned to a large extent by bird ringing [4], with the routes of birds ringed in the breeding grounds and recovered in the winter quarters, or vice-versa, being depicted as straight lines (https://euring.org/research/migration-atlases). These lines create the impression of a single movement between these two homes. The ringing or recovery of birds at points which are intermediate between breeding grounds and winter quarters, where they are shown or assumed to stay for brief periods, has led to the idea of stop-over sites [5]. Stop-over sites are regarded as resting and refuelling stations along the journey. The degree to which migratory birds remain within a single winter home, or move instead between different sites outside the breeding season, has long been recognized [6]. These examples of itinerant behaviour are nevertheless considered as part of species behaviour within the winter quarters. The more recent use of tracking technology has helped to refine this perspective [7, 8]. Here we show, using GPS loggers, that pallid swifts (*Apus pallidus brehmorum*) do not conform to the classical pattern of a bird with two homes but instead, spend varying amounts of time in different geographical areas south of the Sahara Desert, outside the breeding season.

## Materials and methods

A breeding colony of pallid swifts situated in the attic of the Gibraltar National Museum (36.14° N, -5.36° W) was used for the study. This is a well-established colony that was first studied by Finlayson [9]. Adult birds were taken from the nests at night while they were roosting, ringed and tagged, and returned to the nest shortly after. Wing length and body mass were recorded for each individual. Most birds remained at the nest when returned. A few left the nest but returned shortly after. Birds sitting on eggs or with young chicks were left undisturbed.

**Fig 1:**
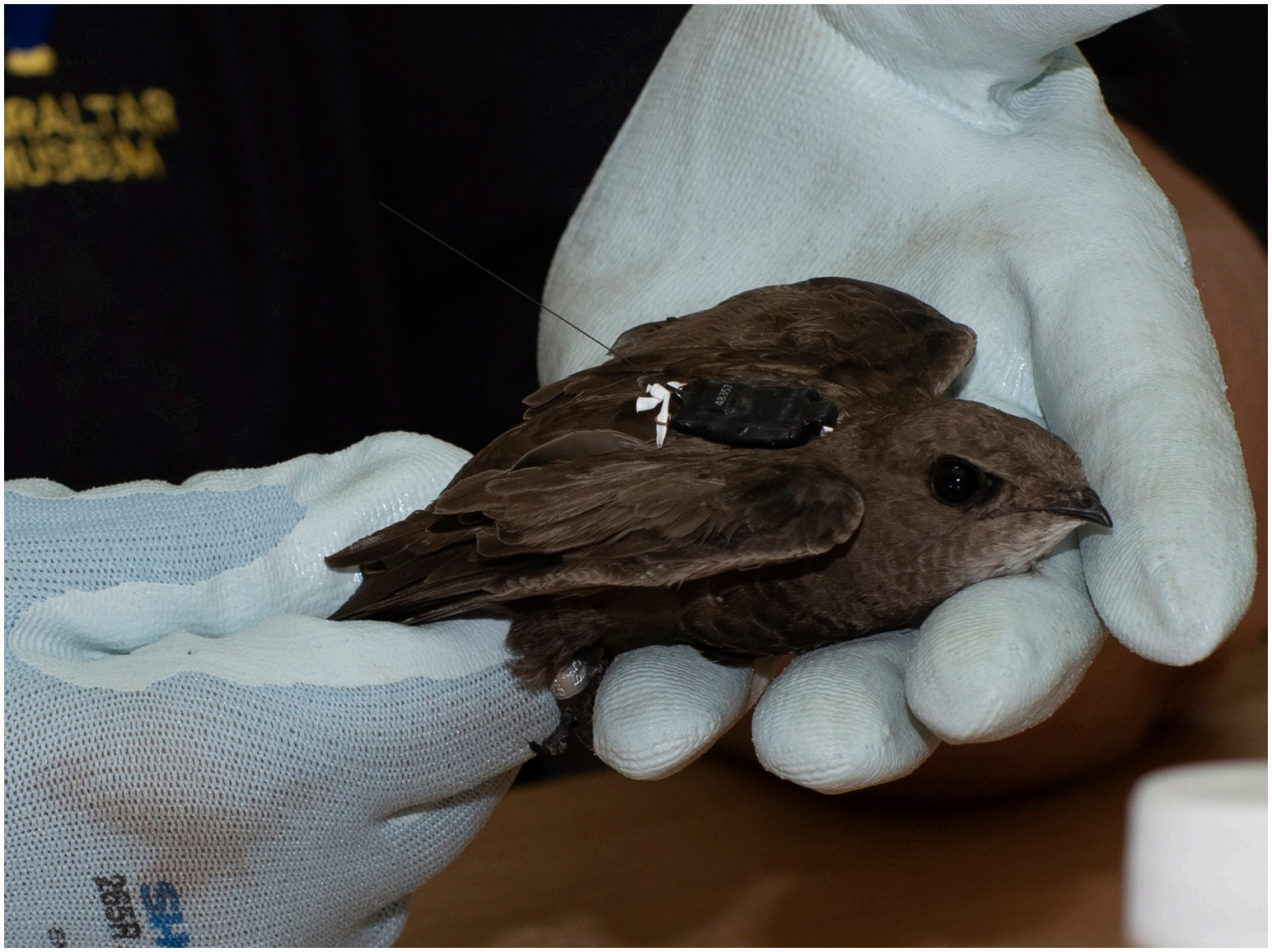
Photograph of pallid swift (Apus pallidus) with GPS tag and harness.

Ten tags were attached to pallid swifts in 2018 and 12 in 2019. Ten (46%) of birds were recovered, seven from 2018 (70%) and three from 2019 (25%). In three cases, all 2018 birds, the tag failed. One individual (from a possible six) was recovered in two consecutive years. Among the birds not recovered were three that did return, detected by infra-red cameras on the nests, but could not be caught. The tags used were GPS store-on-board loggers (model PinPoint 10) manufactured by Lotek/Biotrack, UK (www.lotek.com). The tags were suitable for use on pallid and common swifts (*A*. *apus*) as their weight (31–56 g [10]) does not exceed the 3–5% rule-of-thumb for the relative mass of telemetry or datalogging devices deployed on flying vertebrates [11–13]. Product specifications: weight: 1g; dimensions: 21mm x 13mm x 5mm; antenna: 50mm. Tags were positioned dorsally between the birds’ wings. A full body harness, consisting of a single length of 2mm- wide polytetrafluoroethylene (PTFE) tape, was achieved by tying the centre of the tape to an attachment tube at the front of each tag and looping either end around the neck. The tape was then knotted on the ventral side before passing the ends of the tape under each wing and finally fastening them to each of the two communication loops at the back of the tags (Fig 1). Each tag was set to record at noon (GMT) every four days. In the case of one bird (ring number SB63823), the tag was placed to record four times a day, at 1400, 1700, 2000 and 2300 hours (GMT). The ringing and handling of swifts was carried out under the auspices of the Gibraltar Ornithological and Natural History Society, under licence from the Ministry for the Environment, H. M. Government of Gibraltar, under the 1991 Nature Protection Act. Raw data used in this paper are in S1 Table.

## Results

### Areas used by pallid swifts during the annual cycle

**Fig 2:**
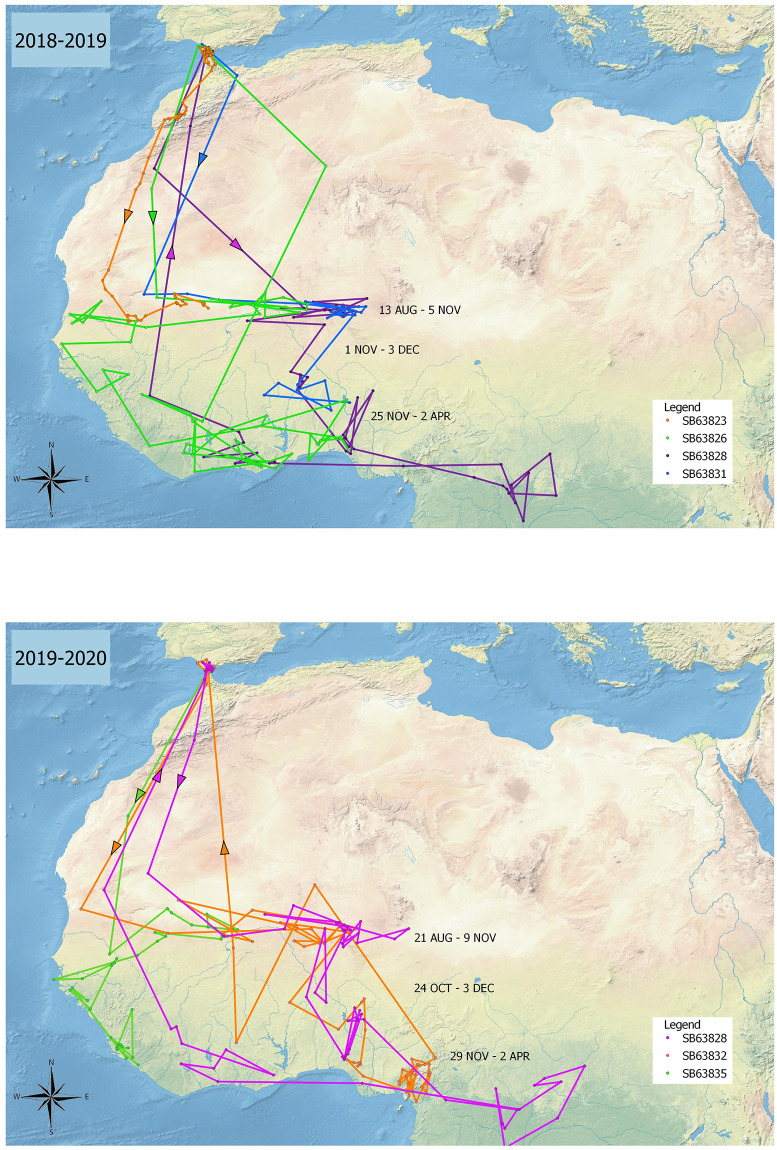
Tracks of routes taken by pallid swifts from the Gibraltar National Museum breeding colony in 2018–19 (four birds) and 2019–20 (three birds). Arrows indicate direction of crossing of the Sahara Desert. Dates indicate limits of time periods spent in the Sahel, Sudanian and Guinea zones.

**Fig 3:**
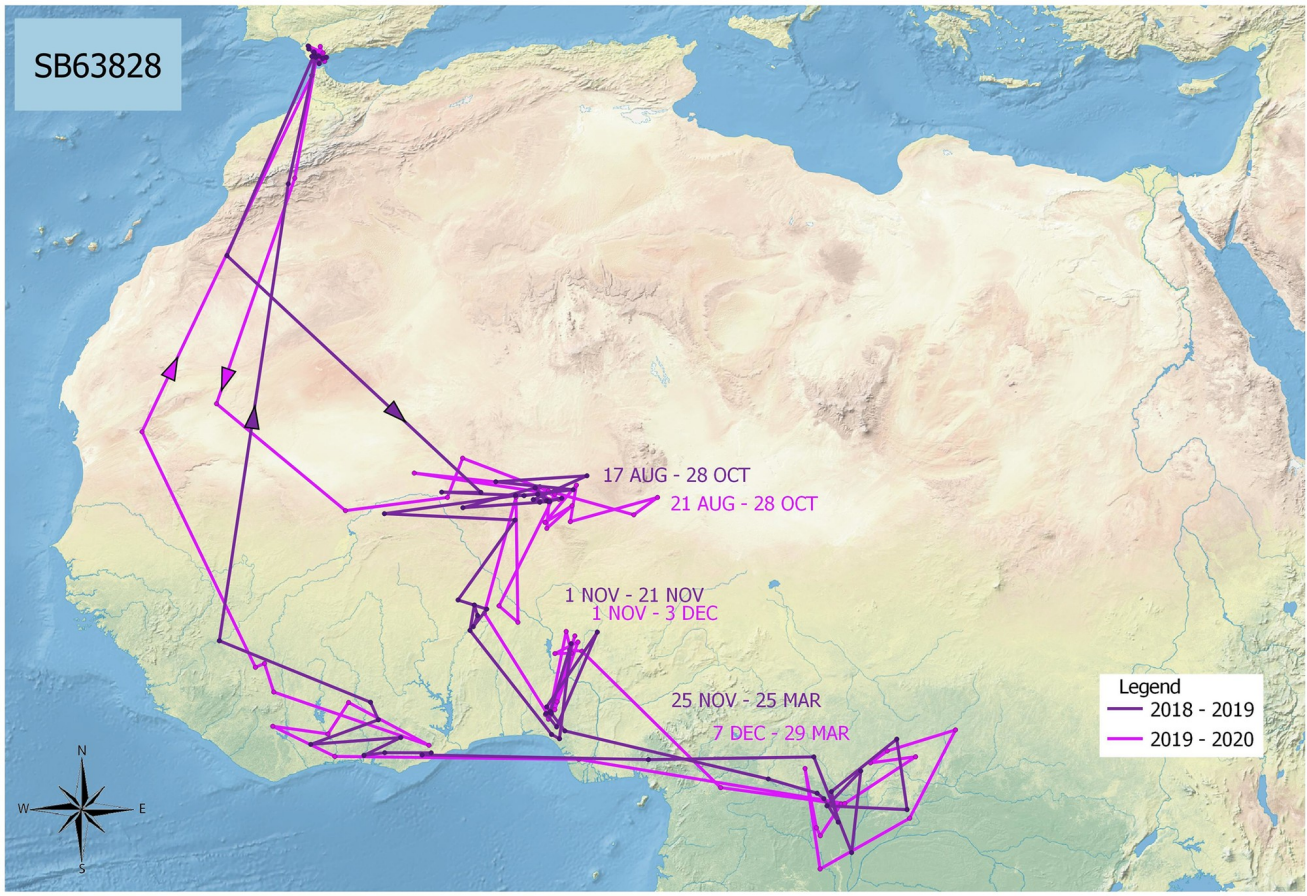
Tracks of routes taken by a single pallid swift (ring number SB63828) from the Gibraltar National Museum breeding colony over two years (2018–19 and 2019–20). Arrows indicate direction of crossing of the Sahara Desert. Dates indicate limits of time periods spent in the Sahel, Sudanian and Guinea zones.

We examined the distribution of pallid swifts by latitude. Pallid swifts from the Gibraltar National Museum breeding colony showed remarkable consistency in phenology in relation to latitude (Fig 2). In both years studied (2018–19 and 2019–20), pallid swifts left the breeding colony in August and, after crossing the Sahara Desert, spent the rest of the month through to the first week in November (around two-and-a-half months) in the Sahel zone (between 15° and 20° N). From there birds spent about one month in the Sudanian savannah zone (between 10° and 15° N), or between this zone and the Sahel to the north, and the Guinean zone to the south, and just over four months in the Guinea forest zone, between 0° and 10° N [14–16]. The single pallid swift (ring number SB63828) which was tracked over two years appeared to show remarkable consistency in the areas visited, and their timing, between years (Fig 3).

**Fig 4:**
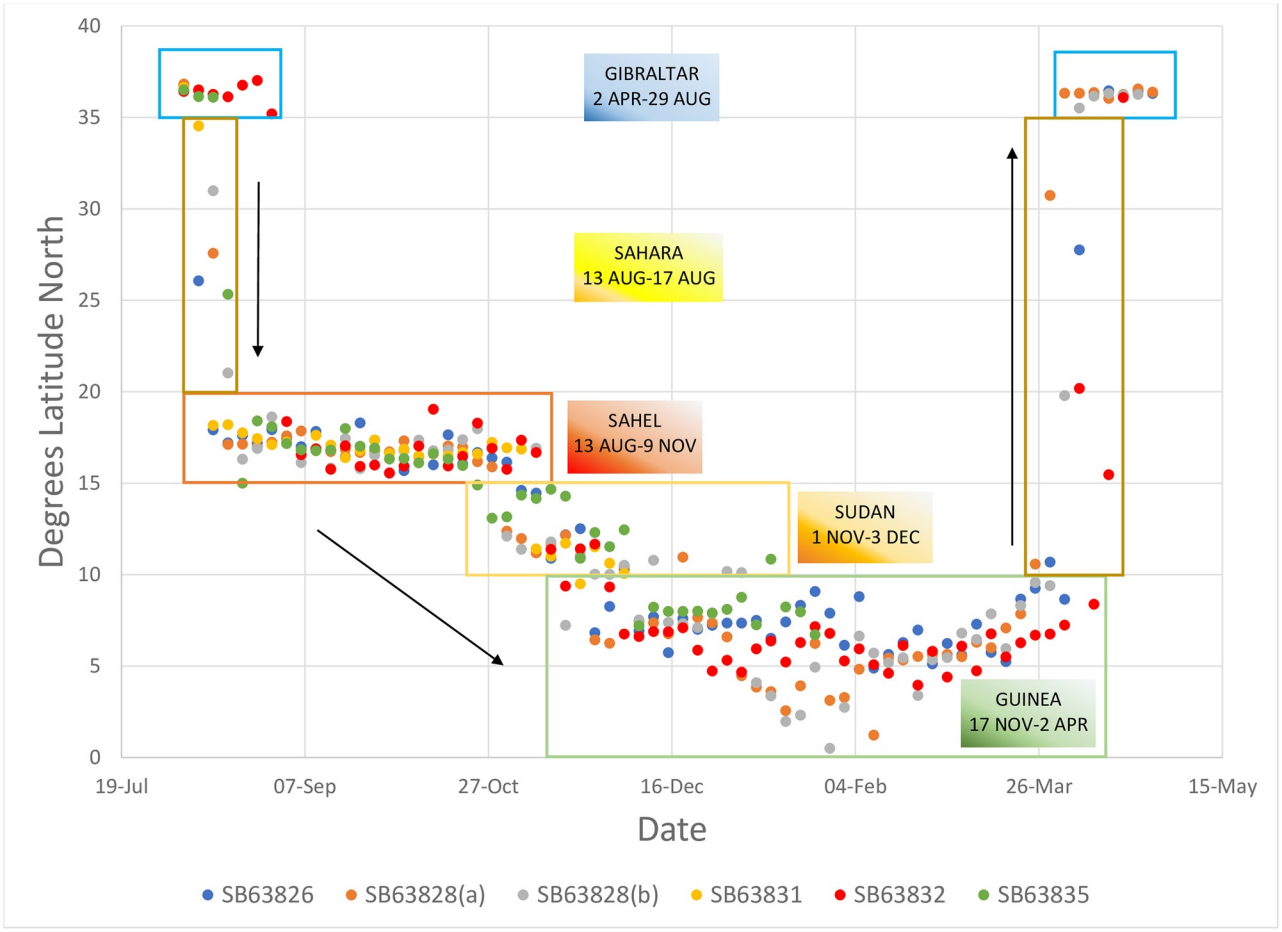
Summary of tracks, by latitude, of routes taken by pallid swifts from the Gibraltar National Museum breeding colony in 2018–19 (four birds) and 2019–20 (three birds). Data from the pallid swift (SB63828) tracked over two years (2018–19 (a); 2019–20 (b)) are included.

**Fig 5:**
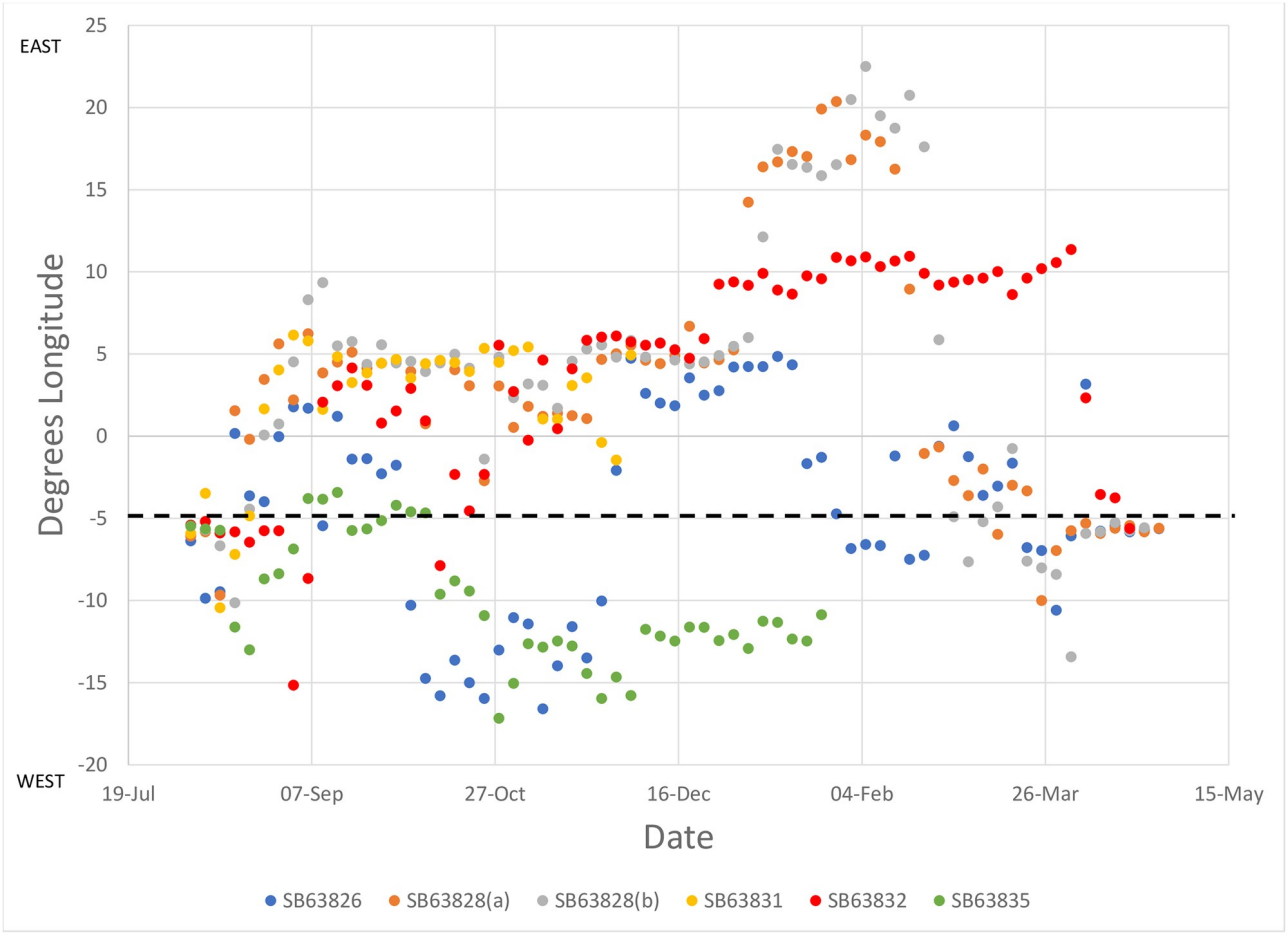
Summary of tracks, by longitude, of routes taken by pallid swifts from the Gibraltar National Museum breeding colony in 2018–19 (four birds) and 2019–20 (three birds). Data from a single pallid swift (SB63828) tracked over two years (2018–19 (a); 2019–20 (b)) are included. Dashed line shows the longitude of the breeding colony at Gibraltar.

**Fig 6:**
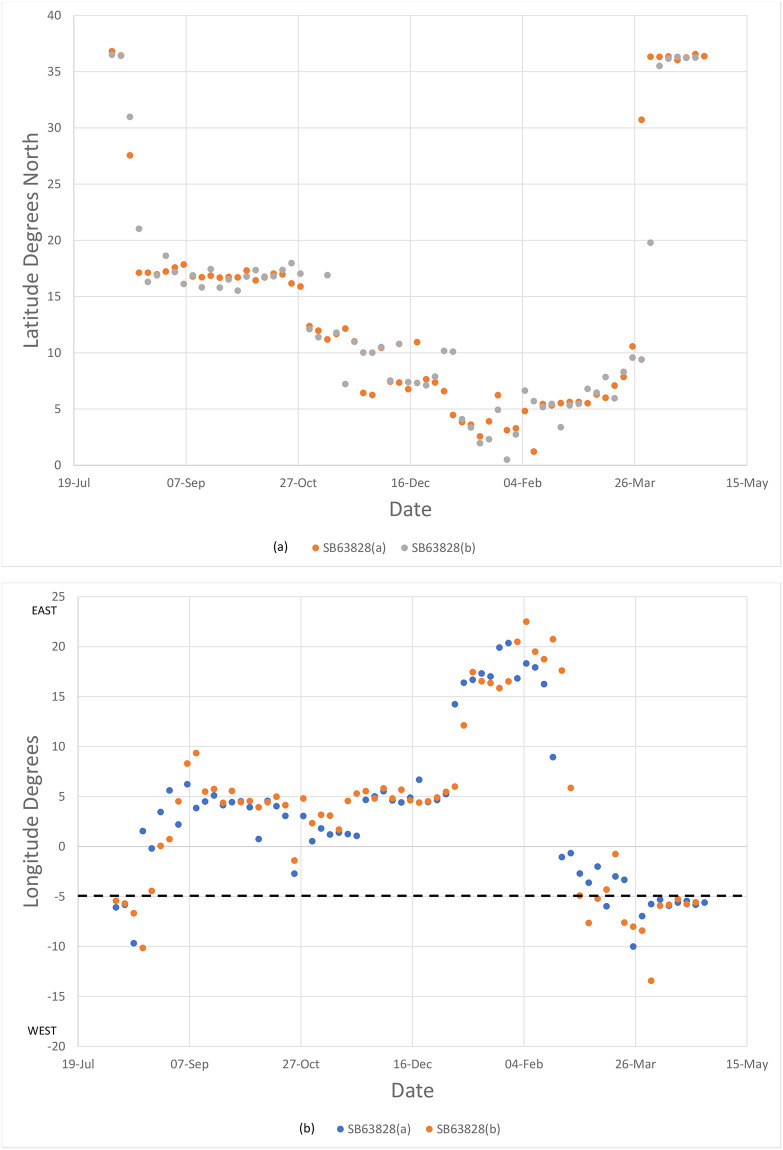
Summary of tracks, by (a) latitude and (b) longitude, of routes taken by a single pallid swift (SB63828) over two years (2018–19 (a); 2019–20 (b)). In (b) dashed line shows the longitude of the breeding colony at Gibraltar.

Pallid swifts occupied latitude bands particular time periods (Fig 4). All pallid swifts were exclusively between 15° and 20° N from mid-August until 1 November, a period of two-and-a-half months. All pallid swifts were exclusively south of 10° N from 3 December until 2 April, a period of four months. Between 1 November until 3 December, the latitude bands occupied overlapped (1–9 November, 15°-20° N with 10°-15° N, and 17 November– 3 December, 10°-25° N with 0°-10° N). Birds were only exclusively between 10° and 15° N just for a week (9–17 November). These results suggest two long periods of occupation of specific latitude bands and a brief one of transition as birds moved from one to the other. This gradual southward change of homes contrasts with the rapid migration across the desert. There was, on the other hand, significant individual longitude variation with regards to the areas visited by pallid swifts (Fig 5). The individual tracked over two years nevertheless, appeared to be broadly consistent, latitudinally and longitudinally, between years (Fig 6).

### Distances covered

**Fig 7:**
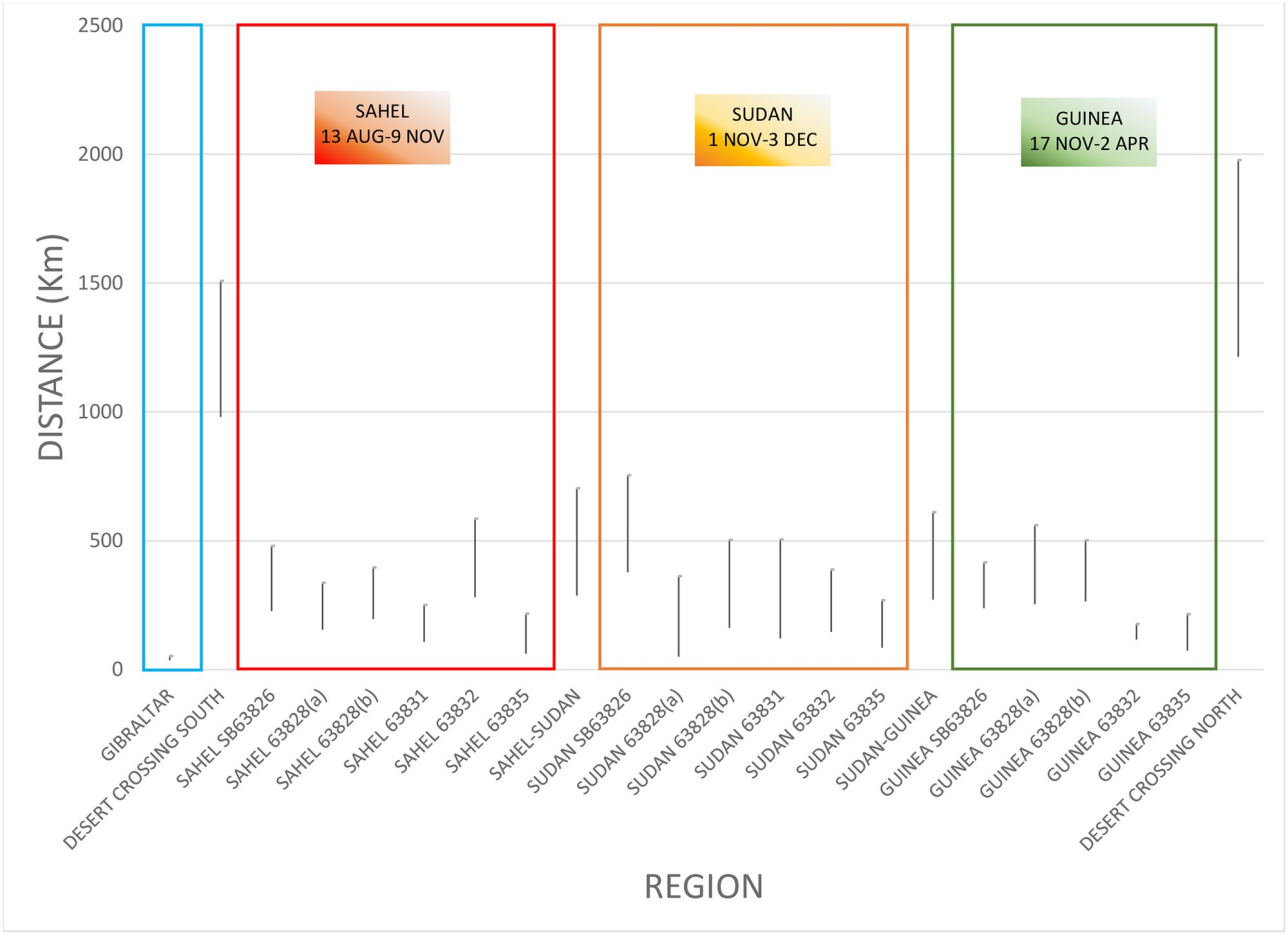
Distances (km/4-days) covered by pallid swifts at different points in the annual cycle. As the last stages at Gibraltar prior to departure, crossing of the Sahara Desert, and between the main zones (Sahel, Sudanian, Gunea) are brief, data from different individuals have been pooled. Within the main areas of the Sahel, Sudanian and Guinea zones, data for individual birds are shown.

Data of speeds achieved by pallid swifts at different points of the annual cycle are summarised as Fig 7. Since readings were taken every four days, it is recognised that data reflect averages at this time interval and birds may have travelled faster or slower at times. The data also assume that each individual travelled between each point in a straight line, which may not have been the case. In spite of these caveats, the results are clear. All data presented are mean with 95% confidence intervals. Pallid swifts at the Gibraltar breeding colony towards the end of the breeding season travelled at relatively slow speeds: 11.1 ± 1.9 (2δ) km/day, while remaining close to the nests. The average speed of travel was greatest when crossing the Sahara Desert: 311.2 ± 65.8 (2δ) km/day, southbound, and 399 ± 95.4 (2δ) km/day, northbound. The northbound crossing appeared to be faster but the differences between both crossings were not statistically significant. It should be noted, however, that individual pallid swifts achieved greater distances in a four-day period when travelling north than when flying south, and a single bird tracked over two years achieved greater average speeds during the northward passage, compared to the southward passage, both years (Table 1). Average speeds achieved during the remaining time, south of the Sahara, were lower than during the crossing, averaging 77 ± 14.1 (2δ) km/day, with no significant differences between the main zones or when travelling between them (Fig 7).

**Table 1:** Maximum distances covered by individual pallid swifts in a four-day interval during southbound and northbound migration across the Sahara Desert.

Ring Number	Start Date	End Date	Distance covered (km)	Migration Direction
SB63826	05/08/18	09/08/18	1236	South
SB63826	02/04/19	06/04/19	2335	North
SB63828	13/08/18	17/08/18	1635	South
SB63828	25/03/19	29/03/19	2263	North
SB63828	13/08/19	17/08/19	1159	South
SB63828	01/04/20	05/04/20	1895	North
SB63832	29/08/19	02/09/19	2088	South
SB63832	13/04/20	17/04/20	2300	North

**Fig 8:**
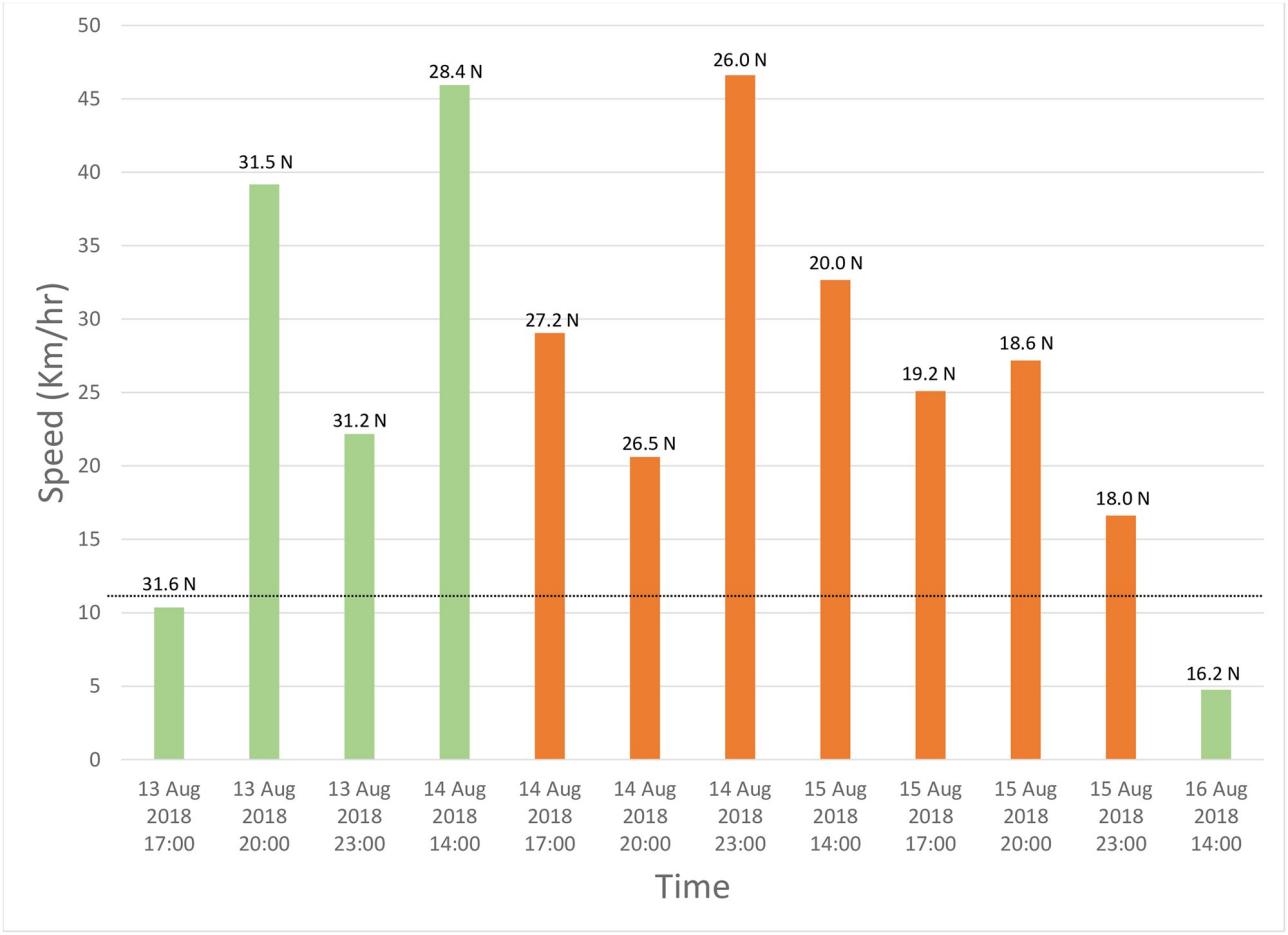
Distances (km/hr) covered by pallid swift SB63823 while crossing the Sahara Desert. Numbers above bars indicate latitude (degrees north) achieved at each particular point. Orange bars indicate that the bird is over the Sahara Desert. Dotted line is the upper 95% confidence limit for all wind speeds achieved by this pallid swift while being tracked (23 July-20 August, 2018).

**Fig 9:**
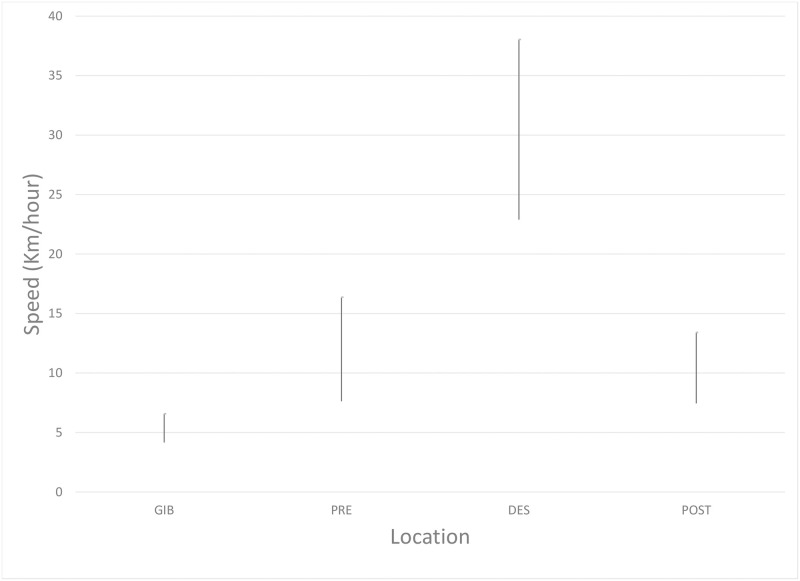
Distances (km/hr) covered by pallid swift SB63823 before, during and after crossing the Sahara Desert. Bars indicate mean ± 95% confidence limits. Data are for period when this pallid swift while being tracked (23 July-20 August, 2018). GIB, Gibraltar; PRE, pre-desert; DES, Sahara Desert; POST, post-desert.

A more accurate estimate of the time taken to cross the Sahara Desert was obtained from one bird (SB63823) which had been tagged to record its position four times a day (see Materials and methods). This bird covered a distance of 1858 km in just over two days, reaching a maximum speed of 46.6 km/hr, with the core of the Sahara being traversed in just over one day: 1700 hours, 14^th^ August—2300 hours, 15^th^ August (Fig 8). This was a non-stop flight, with continuous flying day and night. This individual significantly increased average speed in the build-up to the desert crossing, and once again while over the desert itself (Fig 9).

Two additional pallid swifts were tagged in 2020 to provide data at midnight during the non-breeding season. Data were collected every four days, alternating between midnight and midday. Both birds were in the air on all occasions when readings were taken: SB63835 between 8^th^ August, 2020 and 13^th^ April, 2021, and SB63853 between 18^th^ August, 2020 and 14^th^ March, 2021 (S2 Table).

## Discussion

Our results show that pallid swifts occupy several areas during their annual cycle. After the breeding season they spent two-and-a-half months (mid-August—end October) in the Sahel and four months (December—April) in the Guinean zone, with a month of transition between the two zones (Sahel / Sudan / Guinean zones). This seasonal distribution would appear to be related to the latitudinal influence on tropical African climate, particularly rainfall [14]. The length of stay in the Guinean zone is no greater than that in the breeding area., while the length of stay in the Sahel is around a month-and-a-half shorter. In the sense of having multiple homes, each at specific times of the year, the behaviour of pallid swifts resembles that of some seabird species [17–20]. Although there appears to be a strict adherence to specific latitude bands at specific times of the annual cycle, there is also wide longitudinal flexibility, which is remarkable considering the birds studied came from a single breeding colony. Also in this aspect of individual choice of areas visited, pallid swifts resemble some seabirds [21].

In a study of pallid swifts using light-level geolocators, Hedenström *et al*. [22] considered that birds recorded in November were still on migration. The more-detailed picture provided by GPS loggers shows that these birds would have been, in fact, either in their Sahel home or transiting to the Sudanian savannah home. The speed of travel between these homes was significantly lower than during the migration across the Sahara Desert. Isenmann *et al*. [23] reported high numbers of pallid swifts (up to 10,000/day) on 45 days between 19 August and 19 October, 2003, on the coast of Mauritania, 40 km south of Nouakchott (peak numbers 3–27 September), which they attributed to migration. Rather than migration, we suggest that such numbers reflect concentrations of birds in the Sahel and desert edge in the period August-November. The European population of pallid swifts had been estimated at 24,717 (21,202–32,893) breeding pairs [24] but this seems a significant underestimate as the breeding population in Spain alone is estimated at 40,000 breeding pairs [25]. The number of pallid swifts migrating along the western Sahara could therefore reasonably be expected to be of the order of 100,000 birds, possibly larger if we include north African birds. Even so, the estimate of passage of up to 10,000 birds over a period of 45 days, in a particular point of that region, therefore seems excessive and further suggests that what was being observed were local concentrations of birds in the Sahel/desert edge and not migration.

Hedenström’s [22] three pallid swifts from Carmagnola, Italy, at 44.86°N, 7.72°E, were all west of 5°W in November, at latitudes ranging between 4° and 20°N (mostly north of 10°N). These birds therefore undertook a significant westward shift from the breeding grounds, further emphasizing the importance of the western Sahel for pallid swifts. While one of Hedenström’s [22] pallid swifts remained west of the Greenwich meridian (Cote d’Ivoire), the other two had moved significantly eastwards (to ~12°E), up to Cameroon by February and most records were south of 10°N. A similar ESE shift appears with “late” Pallid Swifts studied by Norevik *et al* [26] compared to those earlier in the season. The behaviour of these birds is consistent with that of the Gibraltar birds and suggests a common pattern of movement by birds from colonies approximately 1,500 km apart.

Pallid swifts from the Gibraltar National Museum colony therefore migrated south in August and remained in the Sahel until early November, when they moved south into the Sudanian savannah zone, where they stayed for approximately one month, before moving into the Guinea forest zone, where they remained until their northward migration back to the breeding colony in late March-early April. This movement within tropical Africa outside the breeding season may explain confusion that has existed with regard to the “wintering” area of this species, particularly with regard to the Sahel-Guinean dichotomy [6, 10, 27]. The Sahel zone is an important area for pallid swifts between August and early November, as it is for other insectivores [28]. The subsequent movement into the Sudanian and, eventually, Guinean zones matches that of other aerial insectivores [29].

Similar movement patterns have been observed in respect of common cuckoos (*Cuculus canorus*), red-backed shrikes (*Lanius collurio*) thrush nightingales (*Luscinia luscinia*) [30] and Montagu’s harriers (*Circus pygargus*) [31] during the non-breeding season in tropical and equatorial Africa. New technology is now revealing the diversity of migrant strategies, from long stationary periods in breeding and wintering grounds (in nightjars, *Caprimulgus europaeus*) to greater mobility between areas (swifts, similar to our pallid swifts) to intermediate strategies (common cuckoo) [32].

Finally, our direct observations, for the first time using GPS tags, of pallid swifts at night in tropical Africa support earlier results obtained using light level geolocators [22]. Whilst impossible to completely disregard landing at some stage, they do indicate a high probability that the birds were largely airborne at night.

## Supporting information

S1 Table Raw data obtained in this study and used in this paper. (DOCX)

S2 Table Data of two pallid swifts during the 2020–21 non-breeding season. Data show that the birds remained airborne throughout, including at night.
(DOCX)
