# Supplementary material for: Birds with multiple homes. The annual cycle of the pallid swift (Apus pallidus brehmorum)
Source: PLoS One. 2021 Nov 30;16(11):e0259656. doi: 10.1371/journal.pone.0259656 (PMC8631615; doi:10.1371/journal.pone.0259656)
Supplement: S1 Table — (DOCX) [file pone.0259656.s001.docx]

S1 Table. Raw data obtained in this study and used in this paper.

| **Ring No.** | **Date** | **Time** | **Latitude** | **Longitude** |
| --- | --- | --- | --- | --- |
| SB63823 | 23/07/2018 | 17:00:08 | NOT ENOUGH SATS | |
| SB63823 | 23/07/2018 | 20:00:06 | NOT ENOUGH SATS | |
| SB63823 | 23/07/2018 | 23:00:06 | 36.10321 | -5.38694 |
| SB63823 | 24/07/2018 | 14:00:04 | 36.22523 | -5.31913 |
| SB63823 | 24/07/2018 | 17:00:05 | 36.27235 | -5.34988 |
| SB63823 | 24/07/2018 | 20:00:11 | 36.05111 | -5.50272 |
| SB63823 | 24/07/2018 | 23:00:06 | 36.08103 | -5.41899 |
| SB63823 | 25/07/2018 | 14:00:10 | 36.24688 | -5.70897 |
| SB63823 | 25/07/2018 | 17:00:05 | 36.18032 | -5.67373 |
| SB63823 | 25/07/2018 | 20:00:11 | 36.18887 | -5.37259 |
| SB63823 | 25/07/2018 | 23:00:04 | 36.09441 | -5.40385 |
| SB63823 | 26/07/2018 | 14:00:07 | 36.5543 | -5.5497 |
| SB63823 | 26/07/2018 | 17:00:07 | 36.3344 | -5.57627 |
| SB63823 | 26/07/2018 | 20:00:11 | 36.27195 | -5.40857 |
| SB63823 | 26/07/2018 | 23:00:07 | 36.10295 | -5.39862 |
| SB63823 | 27/07/2018 | 14:00:11 | 36.24506 | -5.71808 |
| SB63823 | 27/07/2018 | 17:00:04 | 36.21923 | -5.70767 |
| SB63823 | 27/07/2018 | 20:00:04 | 36.21631 | -5.55022 |
| SB63823 | 27/07/2018 | 23:00:07 | 36.04009 | -5.4413 |
| SB63823 | 28/07/2018 | 14:00:05 | 36.22457 | -5.69733 |
| SB63823 | 28/07/2018 | 17:00:06 | 36.08136 | -5.56305 |
| SB63823 | 28/07/2018 | 20:00:05 | 36.11014 | -5.46471 |
| SB63823 | 28/07/2018 | 23:00:09 | 36.05788 | -5.41437 |
| SB63823 | 29/07/2018 | 14:00:08 | 36.33907 | -5.70556 |
| SB63823 | 29/07/2018 | 17:00:08 | 36.34552 | -5.6857 |
| SB63823 | 29/07/2018 | 20:00:11 | 36.20014 | -5.53653 |
| SB63823 | 29/07/2018 | 23:00:06 | 36.1276 | -5.2776 |
| SB63823 | 30/07/2018 | 14:00:11 | 36.25005 | -5.72034 |
| SB63823 | 30/07/2018 | 17:00:11 | 36.3854 | -5.64129 |
| SB63823 | 30/07/2018 | 20:00:11 | 36.20601 | -5.52683 |
| SB63823 | 30/07/2018 | 23:00:08 | 36.05439 | -5.42412 |
| SB63823 | 31/07/2018 | 14:00:04 | 36.2041 | -5.68453 |
| SB63823 | 31/07/2018 | 17:00:05 | 36.19993 | -5.75992 |
| SB63823 | 31/07/2018 | 20:00:06 | 36.12945 | -5.71804 |
| SB63823 | 31/07/2018 | 23:00:10 | 36.04754 | -5.73024 |
| SB63823 | 01/08/2018 | 14:00:05 | 36.22265 | -5.90858 |
| SB63823 | 01/08/2018 | 17:00:05 | 36.23321 | -5.95373 |
| SB63823 | 01/08/2018 | 20:00:11 | 36.19165 | -6.01847 |
| SB63823 | 01/08/2018 | 23:00:11 | NOT ENOUGH SATS | |
| SB63823 | 02/08/2018 | 14:00:07 | 36.58821 | -6.23358 |
| SB63823 | 02/08/2018 | 17:00:05 | 35.97615 | -5.82476 |
| SB63823 | 02/08/2018 | 20:00:04 | 35.46616 | -5.60009 |
| SB63823 | 02/08/2018 | 23:00:07 | 35.33905 | -5.5833 |
| SB63823 | 03/08/2018 | 14:00:04 | 35.51023 | -5.89787 |
| SB63823 | 03/08/2018 | 17:00:04 | 36.12004 | -5.96051 |
| SB63823 | 03/08/2018 | 20:00:04 | 36.14909 | -5.72117 |
| SB63823 | 03/08/2018 | 23:00:08 | 36.04504 | -5.69054 |
| SB63823 | 04/08/2018 | 14:00:10 | 36.1048 | -5.73946 |
| SB63823 | 04/08/2018 | 17:00:05 | 36.11776 | -5.71661 |
| SB63823 | 04/08/2018 | 20:00:04 | 36.12014 | -5.46261 |
| SB63823 | 04/08/2018 | 23:00:09 | 36.0943 | -5.69679 |
| SB63823 | 05/08/2018 | 14:00:06 | 36.26684 | -6.08392 |
| SB63823 | 05/08/2018 | 17:00:04 | 36.1933 | -6.00827 |
| SB63823 | 05/08/2018 | 20:00:04 | 35.95089 | -5.69163 |
| SB63823 | 05/08/2018 | 23:00:05 | 36.11252 | -5.88825 |
| SB63823 | 06/08/2018 | 14:00:07 | 36.64178 | -5.91347 |
| SB63823 | 06/08/2018 | 17:00:07 | 36.58698 | -5.67246 |
| SB63823 | 06/08/2018 | 20:00:04 | 36.49263 | -5.67855 |
| SB63823 | 06/08/2018 | 23:00:05 | 36.15318 | -5.68082 |
| SB63823 | 07/08/2018 | 14:00:05 | 36.28701 | -5.8899 |
| SB63823 | 07/08/2018 | 17:00:09 | 36.2025 | -5.87919 |
| SB63823 | 07/08/2018 | 20:00:04 | 36.25736 | -5.76811 |
| SB63823 | 07/08/2018 | 23:00:04 | 36.01166 | -5.69161 |
| SB63823 | 08/08/2018 | 14:00:04 | 36.24086 | -5.73259 |
| SB63823 | 08/08/2018 | 17:00:11 | 36.26796 | -5.71066 |
| SB63823 | 08/08/2018 | 20:00:04 | 36.31068 | -5.6293 |
| SB63823 | 08/08/2018 | 23:00:04 | 36.04771 | -5.46734 |
| SB63823 | 09/08/2018 | 14:00:06 | 35.34536 | -5.10811 |
| SB63823 | 09/08/2018 | 17:00:09 | 35.38295 | -5.09463 |
| SB63823 | 09/08/2018 | 20:00:05 | 35.13364 | -5.32307 |
| SB63823 | 09/08/2018 | 23:00:05 | 34.86498 | -5.40838 |
| SB63823 | 10/08/2018 | 14:00:06 | 32.49576 | -7.55098 |
| SB63823 | 10/08/2018 | 17:00:04 | 32.13818 | -7.27494 |
| SB63823 | 10/08/2018 | 20:00:04 | 31.87046 | -7.2953 |
| SB63823 | 10/08/2018 | 23:00:04 | 31.54925 | -7.62814 |
| SB63823 | 11/08/2018 | 14:00:04 | 31.21148 | -7.93263 |
| SB63823 | 11/08/2018 | 17:00:04 | 31.59722 | -7.68484 |
| SB63823 | 11/08/2018 | 20:00:04 | 31.25449 | -8.49866 |
| SB63823 | 11/08/2018 | 23:00:04 | 31.29984 | -8.61755 |
| SB63823 | 12/08/2018 | 14:00:11 | 31.39518 | -7.77478 |
| SB63823 | 12/08/2018 | 17:00:05 | 31.65848 | -7.78056 |
| SB63823 | 12/08/2018 | 20:00:04 | 31.55059 | -7.7087 |
| SB63823 | 12/08/2018 | 23:00:04 | 31.41009 | -7.81676 |
| SB63823 | 13/08/2018 | 14:00:10 | 31.61912 | -7.40103 |
| SB63823 | 13/08/2018 | 17:00:04 | 31.61943 | -7.40241 |
| SB63823 | 13/08/2018 | 20:00:04 | 31.54091 | -7.71718 |
| SB63823 | 13/08/2018 | 23:00:04 | 31.19626 | -8.88709 |
| SB63823 | 14/08/2018 | 14:00:06 | 28.38899 | -10.0756 |
| SB63823 | 14/08/2018 | 17:00:03 | 27.20216 | -10.4795 |
| SB63823 | 14/08/2018 | 20:00:04 | 26.44882 | -10.7215 |
| SB63823 | 14/08/2018 | 23:00:04 | 25.97393 | -11.0438 |
| SB63823 | 15/08/2018 | 14:00:04 | 19.97384 | -13.0828 |
| SB63823 | 15/08/2018 | 17:00:04 | 19.19895 | -13.528 |
| SB63823 | 15/08/2018 | 20:00:04 | 18.54684 | -13.3355 |
| SB63823 | 15/08/2018 | 23:00:05 | 18.02012 | -12.7984 |
| SB63823 | 16/08/2018 | 14:00:04 | 16.16165 | -11.4876 |
| SB63823 | 16/08/2018 | 17:00:04 | 16.11214 | -11.6107 |
| SB63823 | 16/08/2018 | 20:00:04 | 16.59778 | -11.634 |
| SB63823 | 16/08/2018 | 23:00:04 | 16.5721 | -11.6485 |
| SB63823 | 17/08/2018 | 14:00:07 | 16.53686 | -11.064 |
| SB63823 | 17/08/2018 | 17:00:05 | 16.22803 | -10.8744 |
| SB63823 | 17/08/2018 | 20:00:04 | 16.20651 | -10.6561 |
| SB63823 | 17/08/2018 | 23:00:04 | 16.54728 | -10.3172 |
| SB63823 | 18/08/2018 | 14:00:04 | 17.56415 | -8.02252 |
| SB63823 | 18/08/2018 | 17:00:04 | 17.45576 | -7.95893 |
| SB63823 | 18/08/2018 | 20:00:04 | 17.48914 | -7.92887 |
| SB63823 | 18/08/2018 | 23:00:04 | 17.30076 | -7.68386 |
| SB63823 | 19/08/2018 | 14:00:04 | 17.43095 | -7.28142 |
| SB63823 | 19/08/2018 | 17:00:05 | 17.65102 | -7.54669 |
| SB63823 | 19/08/2018 | 20:00:06 | 17.96949 | -8.10239 |
| SB63823 | 19/08/2018 | 23:00:04 | 18.20218 | -8.16882 |
| SB63823 | 20/08/2018 | 14:00:04 | 17.47984 | -5.91951 |
| SB63823 | 20/08/2018 | 17:00:04 | 17.32429 | -5.98805 |
| SB63823 | 20/08/2018 | 20:00:04 | 17.48172 | -6.04912 |
| SB63823 | 20/08/2018 | 23:00:05 | 17.12342 | -5.6272 |
|  |  |  |  |  |
| SB63826 | 05/08/2018 | 12:00:11 | 36.77012 | -6.36947 |
| SB63826 | 09/08/2018 | 12:00:04 | 26.05757 | -9.85779 |
| SB63826 | 13/08/2018 | 12:00:04 | 17.91356 | -9.46736 |
| SB63826 | 17/08/2018 | 12:00:04 | 17.20253 | 0.1676 |
| SB63826 | 21/08/2018 | 12:00:04 | 17.60286 | -3.62307 |
| SB63826 | 25/08/2018 | 12:00:04 | 17.19319 | -3.98028 |
| SB63826 | 29/08/2018 | 12:00:05 | 17.93085 | -0.02754 |
| SB63826 | 02/09/2018 | 12:00:06 | 17.42604 | 1.78495 |
| SB63826 | 06/09/2018 | 12:00:05 | 16.99331 | 1.70727 |
| SB63826 | 10/09/2018 | 12:00:04 | 17.82273 | -5.44637 |
| SB63826 | 14/09/2018 | 12:00:06 | 16.80435 | 1.20649 |
| SB63826 | 18/09/2018 | 12:00:06 | 16.44217 | -1.39829 |
| SB63826 | 22/09/2018 | 12:00:08 | 18.29466 | -1.35937 |
| SB63826 | 26/09/2018 | 12:00:04 | 17.3532 | -2.28838 |
| SB63826 | 30/09/2018 | 12:00:05 | 16.67858 | -1.76401 |
| SB63826 | 04/10/2018 | 12:00:10 | 15.68946 | -10.2912 |
| SB63826 | 08/10/2018 | 12:00:04 | 16.46679 | -14.7426 |
| SB63826 | 12/10/2018 | 12:00:04 | 16.00199 | -15.7963 |
| SB63826 | 16/10/2018 | 12:00:05 | 17.64834 | -13.6248 |
| SB63826 | 20/10/2018 | 12:00:04 | 16.05242 | -14.9928 |
| SB63826 | 24/10/2018 | 12:00:04 | 16.67815 | -15.9643 |
| SB63826 | 28/10/2018 | 12:00:04 | 16.38618 | -13.008 |
| SB63826 | 01/11/2018 | 12:00:04 | 16.15442 | -11.0367 |
| SB63826 | 05/11/2018 | 12:00:04 | 14.60104 | -11.4185 |
| SB63826 | 09/11/2018 | 12:00:05 | 14.45581 | -16.584 |
| SB63826 | 13/11/2018 | 12:00:04 | 10.89128 | -13.9644 |
| SB63826 | 17/11/2018 | 12:00:04 | 12.17988 | -11.585 |
| SB63826 | 21/11/2018 | 12:00:05 | 12.51484 | -13.4976 |
| SB63826 | 25/11/2018 | 12:00:06 | 6.81946 | -10.0228 |
| SB63826 | 29/11/2018 | 12:00:04 | 8.25197 | -2.07239 |
| SB63826 | 03/12/2018 | 12:00:08 | 10.27471 | 4.74518 |
| SB63826 | 07/12/2018 | 12:00:04 | 6.90943 | 2.60374 |
| SB63826 | 11/12/2018 | 12:00:05 | 7.69424 | 2.01896 |
| SB63826 | 15/12/2018 | 12:00:05 | 5.73316 | 1.85171 |
| SB63826 | 19/12/2018 | 12:00:04 | 7.60055 | 3.5559 |
| SB63826 | 23/12/2018 | 12:00:04 | 7.00879 | 2.50495 |
| SB63826 | 27/12/2018 | 12:00:04 | 7.22521 | 2.76995 |
| SB63826 | 31/12/2018 | 12:00:04 | 7.35102 | 4.209 |
| SB63826 | 04/01/2019 | 12:00:04 | 7.3525 | 4.23989 |
| SB63826 | 08/01/2019 | 12:00:11 | 7.49786 | 4.23496 |
| SB63826 | 12/01/2019 | 12:00:04 | 6.5149 | 4.85068 |
| SB63826 | 16/01/2019 | 12:00:04 | 7.40932 | 4.35464 |
| SB63826 | 20/01/2019 | 12:00:04 | 8.3234 | -1.66859 |
| SB63826 | 24/01/2019 | 12:00:04 | 9.08135 | -1.28784 |
| SB63826 | 28/01/2019 | 12:00:04 | 7.89279 | -4.72396 |
| SB63826 | 01/02/2019 | 12:00:07 | 6.14265 | -6.82789 |
| SB63826 | 05/02/2019 | 12:00:05 | 8.79702 | -6.58906 |
| SB63826 | 09/02/2019 | 12:00:05 | 4.8899 | -6.64904 |
| SB63826 | 13/02/2019 | 12:00:04 | 5.63628 | -1.20278 |
| SB63826 | 17/02/2019 | 12:00:05 | 6.28956 | -7.49701 |
| SB63826 | 21/02/2019 | 12:00:06 | 6.9747 | -7.24478 |
| SB63826 | 25/02/2019 | 12:00:05 | 5.12113 | -0.60757 |
| SB63826 | 01/03/2019 | 12:00:04 | 6.24167 | 0.63226 |
| SB63826 | 05/03/2019 | 12:00:04 | 5.60333 | -1.23843 |
| SB63826 | 09/03/2019 | 12:00:04 | 7.29227 | -3.59631 |
| SB63826 | 13/03/2019 | 12:00:08 | 5.74849 | -3.04291 |
| SB63826 | 17/03/2019 | 12:00:04 | 5.23958 | -1.63534 |
| SB63826 | 21/03/2019 | 12:00:04 | 8.65983 | -6.77971 |
| SB63826 | 25/03/2019 | 12:00:06 | 9.2524 | -6.95882 |
| SB63826 | 29/03/2019 | 12:00:04 | 10.68419 | -10.5819 |
| SB63826 | 02/04/2019 | 12:00:04 | 8.64955 | -6.06533 |
| SB63826 | 06/04/2019 | 12:00:06 | 27.7547 | 3.17261 |
| SB63826 | 10/04/2019 | 12:00:10 | 36.33151 | -5.77406 |
| SB63826 | 14/04/2019 | 12:00:04 | 36.45364 | -5.44561 |
| SB63826 | 18/04/2019 | 12:00:10 | 36.18423 | -5.81415 |
| SB63826 | 22/04/2019 | 12:00:05 | 36.42863 | -5.70172 |
| SB63826 | 26/04/2019 | 12:00:11 | 36.30953 | -5.62279 |
|  |  |  |  |  |
| SB63828 | 05/08/2018 | 12:00:11 | 36.81978 | -6.08319 |
| SB63828 | 09/08/2018 | 12:00:09 | 36.43959 | -5.81959 |
| SB63828 | 13/08/2018 | 12:00:04 | 27.56748 | -9.66775 |
| SB63828 | 17/08/2018 | 12:00:05 | 17.12031 | 1.54636 |
| SB63828 | 21/08/2018 | 12:00:04 | 17.12933 | -0.18621 |
| SB63828 | 25/08/2018 | 12:00:07 | 16.97298 | 3.45362 |
| SB63828 | 29/08/2018 | 12:00:08 | 17.23686 | 5.62611 |
| SB63828 | 02/09/2018 | 12:00:08 | 17.58788 | 2.20497 |
| SB63828 | 06/09/2018 | 12:00:04 | 17.85259 | 6.23286 |
| SB63828 | 10/09/2018 | 12:00:04 | 16.79916 | 3.86125 |
| SB63828 | 14/09/2018 | 12:00:06 | 16.72071 | 4.51299 |
| SB63828 | 18/09/2018 | 12:00:04 | 16.85807 | 5.12131 |
| SB63828 | 22/09/2018 | 12:00:06 | 16.68151 | 4.14685 |
| SB63828 | 26/09/2018 | 12:00:06 | 16.74633 | 4.44792 |
| SB63828 | 30/09/2018 | 12:00:06 | 16.71274 | 4.54074 |
| SB63828 | 04/10/2018 | 12:00:06 | 17.31611 | 3.94256 |
| SB63828 | 08/10/2018 | 12:00:04 | 16.44204 | 0.75318 |
| SB63828 | 12/10/2018 | 12:00:05 | 16.71455 | 4.58546 |
| SB63828 | 16/10/2018 | 12:00:04 | 17.03482 | 4.0508 |
| SB63828 | 20/10/2018 | 12:00:04 | 16.97894 | 3.07869 |
| SB63828 | 24/10/2018 | 12:00:05 | 16.1799 | -2.71499 |
| SB63828 | 28/10/2018 | 12:00:05 | 15.89674 | 3.05932 |
| SB63828 | 01/11/2018 | 12:00:04 | 12.36977 | 0.53711 |
| SB63828 | 05/11/2018 | 12:00:08 | 11.96659 | 1.8069 |
| SB63828 | 09/11/2018 | 12:00:04 | 11.19054 | 1.21374 |
| SB63828 | 13/11/2018 | 12:00:04 | 11.68483 | 1.40066 |
| SB63828 | 17/11/2018 | 12:00:06 | 12.15063 | 1.25736 |
| SB63828 | 21/11/2018 | 12:00:05 | 11.02586 | 1.06742 |
| SB63828 | 25/11/2018 | 12:00:04 | 6.43673 | 4.67046 |
| SB63828 | 29/11/2018 | 12:00:06 | 6.24653 | 5.01761 |
| SB63828 | 03/12/2018 | 12:00:07 | 10.44437 | 5.5372 |
| SB63828 | 07/12/2018 | 12:00:04 | 7.44229 | 4.61799 |
| SB63828 | 11/12/2018 | 12:00:05 | 7.36187 | 4.42026 |
| SB63828 | 15/12/2018 | 12:00:05 | 6.76864 | 4.89206 |
| SB63828 | 19/12/2018 | 12:00:05 | 10.95399 | 6.68935 |
| SB63828 | 23/12/2018 | 12:00:08 | 7.65187 | 4.45153 |
| SB63828 | 27/12/2018 | 12:00:04 | 7.36649 | 4.65986 |
| SB63828 | 31/12/2018 | 12:00:04 | 6.59417 | 5.25307 |
| SB63828 | 04/01/2019 | 12:00:10 | 4.46994 | 14.24309 |
| SB63828 | 08/01/2019 | 12:00:07 | 3.84264 | 16.39421 |
| SB63828 | 12/01/2019 | 12:00:06 | 3.60169 | 16.69548 |
| SB63828 | 16/01/2019 | 12:00:06 | 2.56536 | 17.32893 |
| SB63828 | 20/01/2019 | 12:00:05 | 3.91519 | 17.02677 |
| SB63828 | 24/01/2019 | 12:00:05 | 6.2309 | 19.91453 |
| SB63828 | 28/01/2019 | 12:00:05 | 3.1176 | 20.37027 |
| SB63828 | 01/02/2019 | 12:00:04 | 3.28496 | 16.83148 |
| SB63828 | 05/02/2019 | 12:00:05 | 4.82349 | 18.32259 |
| SB63828 | 09/02/2019 | 12:00:04 | 1.22151 | 17.91947 |
| SB63828 | 13/02/2019 | 12:00:07 | 5.44535 | 16.25148 |
| SB63828 | 17/02/2019 | 12:00:05 | 5.32491 | 8.94869 |
| SB63828 | 21/02/2019 | 12:00:07 | 5.53124 | -1.05195 |
| SB63828 | 25/02/2019 | 12:00:04 | 5.62615 | -0.64673 |
| SB63828 | 01/03/2019 | 12:00:11 | 5.63072 | -2.69832 |
| SB63828 | 05/03/2019 | 12:00:07 | 5.51331 | -3.61708 |
| SB63828 | 09/03/2019 | 12:00:04 | 6.30084 | -1.98982 |
| SB63828 | 13/03/2019 | 12:00:06 | 5.99873 | -5.96624 |
| SB63828 | 17/03/2019 | 12:00:06 | 7.0828 | -2.97886 |
| SB63828 | 21/03/2019 | 12:00:11 | 7.8576 | -3.32614 |
| SB63828 | 25/03/2019 | 12:00:09 | 10.5705 | -10.0027 |
| SB63828 | 29/03/2019 | 12:00:11 | 30.73119 | -6.9593 |
| SB63828 | 02/04/2019 | 12:00:05 | 36.32486 | -5.74663 |
| SB63828 | 06/04/2019 | 12:00:11 | 36.3237 | -5.29844 |
| SB63828 | 10/04/2019 | 12:00:11 | 36.35959 | -5.91972 |
| SB63828 | 14/04/2019 | 12:00:07 | 36.03652 | -5.59441 |
| SB63828 | 18/04/2019 | 12:00:11 | 36.26498 | -5.43611 |
| SB63828 | 22/04/2019 | 12:00:11 | 36.55607 | -5.82017 |
| SB63828 | 26/04/2019 | 12:00:09 | 36.37761 | -5.60557 |
| SB63828 |  |  |  |  |
| SB63828 | 24/07/2019 | 12:00:11 | 36.79794 | -5.54553 |
| SB63828 | 28/07/2019 | 12:00:11 | 36.34782 | -5.70591 |
| SB63828 | 01/08/2019 | 12:00:11 | 36.80378 | -6.05365 |
| SB63828 | 05/08/2019 | 12:00:11 | 36.51509 | -5.41619 |
| SB63828 | 09/08/2019 | 12:00:05 | 36.41568 | -5.69265 |
| SB63828 | 13/08/2019 | 12:00:11 | 30.9881 | -6.67218 |
| SB63828 | 17/08/2019 | 12:00:04 | 21.03045 | -10.1288 |
| SB63828 | 21/08/2019 | 12:00:07 | 16.31368 | -4.43406 |
| SB63828 | 25/08/2019 | 12:00:04 | 16.8963 | 0.07006 |
| SB63828 | 29/08/2019 | 12:00:05 | 18.62829 | 0.74116 |
| SB63828 | 02/09/2019 | 12:00:06 | 17.18351 | 4.52987 |
| SB63828 | 06/09/2019 | 12:00:07 | 16.12954 | 8.30247 |
| SB63828 | 10/09/2019 | 12:00:09 | 16.89637 | 9.34755 |
| SB63828 | 14/09/2019 | 12:00:04 | 15.82617 | 5.49445 |
| SB63828 | 18/09/2019 | 12:00:06 | 17.44062 | 5.7646 |
| SB63828 | 22/09/2019 | 12:00:05 | 15.79621 | 4.37859 |
| SB63828 | 26/09/2019 | 12:00:06 | 16.52918 | 5.57368 |
| SB63828 | 30/09/2019 | 12:00:06 | 15.52874 | 4.45851 |
| SB63828 | 04/10/2019 | 12:00:05 | 16.78486 | 4.5589 |
| SB63828 | 08/10/2019 | 12:00:06 | 17.34981 | 3.94271 |
| SB63828 | 12/10/2019 | 12:00:08 | 16.77544 | 4.45964 |
| SB63828 | 16/10/2019 | 12:00:07 | 16.80932 | 4.99751 |
| SB63828 | 20/10/2019 | 12:00:08 | 17.36946 | 4.14865 |
| SB63828 | 24/10/2019 | 12:00:06 | 17.97162 | -1.39706 |
| SB63828 | 28/10/2019 | 12:00:04 | 17.03767 | 4.81 |
| SB63828 | 01/11/2019 | 12:00:08 | 12.11157 | 2.34924 |
| SB63828 | 05/11/2019 | 12:00:04 | 11.38462 | 3.18171 |
| SB63828 | 09/11/2019 | 12:00:07 | 16.91026 | 3.09795 |
| SB63828 | 13/11/2019 | 12:00:04 | 11.78871 | 1.70051 |
| SB63828 | 17/11/2019 | 12:00:05 | 7.2241 | 4.56068 |
| SB63828 | 21/11/2019 | 12:00:05 | 10.98221 | 5.31336 |
| SB63828 | 25/11/2019 | 12:00:04 | 10.02078 | 5.54967 |
| SB63828 | 29/11/2019 | 12:00:06 | 10.00567 | 4.81603 |
| SB63828 | 03/12/2019 | 12:00:06 | 10.51401 | 5.83176 |
| SB63828 | 07/12/2019 | 12:00:05 | 7.52755 | 4.81345 |
| SB63828 | 11/12/2019 | 12:00:10 | 10.7872 | 5.69696 |
| SB63828 | 15/12/2019 | 12:00:06 | 7.38698 | 4.63557 |
| SB63828 | 19/12/2019 | 12:00:05 | 7.31778 | 4.40195 |
| SB63828 | 23/12/2019 | 12:00:08 | 7.11138 | 4.52991 |
| SB63828 | 27/12/2019 | 12:00:07 | 7.87683 | 4.91228 |
| SB63828 | 31/12/2019 | 12:00:10 | 10.1663 | 5.4664 |
| SB63828 | 04/01/2020 | 12:00:10 | 10.1108 | 6.00875 |
| SB63828 | 08/01/2020 | 12:00:05 | 4.09133 | 12.13292 |
| SB63828 | 12/01/2020 | 12:00:05 | 3.36727 | 17.46113 |
| SB63828 | 16/01/2020 | 12:00:04 | 1.96582 | 16.5363 |
| SB63828 | 20/01/2020 | 12:00:06 | 2.31764 | 16.36472 |
| SB63828 | 24/01/2020 | 12:00:11 | 4.9343 | 15.8629 |
| SB63828 | 28/01/2020 | 12:00:06 | 0.49838 | 16.52641 |
| SB63828 | 01/02/2020 | 12:00:05 | 2.73943 | 20.48168 |
| SB63828 | 05/02/2020 | 12:00:05 | 6.63746 | 22.50832 |
| SB63828 | 09/02/2020 | 12:00:05 | 5.70329 | 19.49523 |
| SB63828 | 13/02/2020 | 12:00:04 | 5.18154 | 18.75315 |
| SB63828 | 17/02/2020 | 12:00:04 | 5.45311 | 20.74564 |
| SB63828 | 21/02/2020 | 12:00:10 | 3.38728 | 17.61661 |
| SB63828 | 25/02/2020 | 12:00:06 | 5.34035 | 5.87064 |
| SB63828 | 29/02/2020 | 12:00:06 | 5.46708 | -4.90443 |
| SB63828 | 04/03/2020 | 12:00:06 | 6.79811 | -7.64337 |
| SB63828 | 08/03/2020 | 12:00:11 | 6.45854 | -5.20295 |
| SB63828 | 12/03/2020 | 12:00:06 | 7.84765 | -4.29659 |
| SB63828 | 16/03/2020 | 12:00:06 | 5.95744 | -0.74505 |
| SB63828 | 20/03/2020 | 12:00:06 | 8.30807 | -7.60201 |
| SB63828 | 24/03/2020 | 12:00:11 | 9.57016 | -8.00951 |
| SB63828 | 28/03/2020 | 12:00:06 | 9.39883 | -8.40891 |
| SB63828 | 01/04/2020 | 12:00:04 | 19.79494 | -13.419 |
| SB63828 | 05/04/2020 | 12:00:12 | 35.51345 | -5.91984 |
| SB63828 | 09/04/2020 | 12:00:10 | 36.15658 | -5.823 |
| SB63828 | 13/04/2020 | 12:00:11 | 36.31336 | -5.26218 |
| SB63828 | 17/04/2020 | 12:00:08 | 36.23167 | -5.74393 |
| SB63828 | 21/04/2020 | 12:00:11 | 36.25791 | -5.5725 |
|  |  |  |  |  |
| SB63831 | 05/08/2018 | 12:00:08 | 36.64312 | -5.91386 |
| SB63831 | 09/08/2018 | 12:00:04 | 34.53009 | -3.47623 |
| SB63831 | 13/08/2018 | 12:00:11 | 18.15616 | -10.4336 |
| SB63831 | 17/08/2018 | 12:00:06 | 18.19565 | -7.18913 |
| SB63831 | 21/08/2018 | 12:00:04 | 17.75381 | -4.84328 |
| SB63831 | 25/08/2018 | 12:00:09 | 17.43951 | 1.66753 |
| SB63831 | 29/08/2018 | 12:00:04 | 17.10974 | 4.02902 |
| SB63831 | 02/09/2018 | 12:00:05 | 17.25437 | 6.15592 |
| SB63831 | 06/09/2018 | 12:00:06 | 16.80444 | 5.79769 |
| SB63831 | 10/09/2018 | 12:00:04 | 17.62052 | 1.63462 |
| SB63831 | 14/09/2018 | 12:00:06 | 17.08411 | 4.83803 |
| SB63831 | 18/09/2018 | 12:00:06 | 16.38883 | 3.26614 |
| SB63831 | 22/09/2018 | 12:00:11 | 16.79069 | 3.85172 |
| SB63831 | 26/09/2018 | 12:00:06 | 17.36077 | 4.44916 |
| SB63831 | 30/09/2018 | 12:00:10 | 16.632 | 4.67269 |
| SB63831 | 04/10/2018 | 12:00:05 | 16.85873 | 3.54566 |
| SB63831 | 08/10/2018 | 12:00:08 | 16.48776 | 4.41879 |
| SB63831 | 12/10/2018 | 12:00:06 | 16.58969 | 4.61542 |
| SB63831 | 16/10/2018 | 12:00:07 | 16.49087 | 4.48928 |
| SB63831 | 20/10/2018 | 12:00:07 | 16.67075 | 3.93283 |
| SB63831 | 24/10/2018 | 12:00:07 | 16.60505 | 5.34444 |
| SB63831 | 28/10/2018 | 12:00:04 | 17.22296 | 4.4993 |
| SB63831 | 01/11/2018 | 12:00:04 | 16.93872 | 5.21551 |
| SB63831 | 05/11/2018 | 12:00:05 | 16.8641 | 5.43523 |
| SB63831 | 09/11/2018 | 12:00:04 | 11.40966 | 1.04995 |
| SB63831 | 13/11/2018 | 12:00:04 | 11.00308 | 1.0255 |
| SB63831 | 17/11/2018 | 12:00:04 | 11.70803 | 3.09311 |
| SB63831 | 21/11/2018 | 12:00:04 | 9.49207 | 3.55478 |
| SB63831 | 25/11/2018 | 12:00:04 | 11.52311 | -0.37751 |
| SB63831 | 29/11/2018 | 12:00:05 | 10.62168 | -1.44872 |
| SB63831 | 03/12/2018 | 12:00:05 | 10.06448 | 4.94359 |
|  |  |  |  |  |
| SB63832 | 24/07/2019 | 12:00:12 | 36.1712 | -5.81336 |
| SB63832 | 28/07/2019 | 12:00:11 | 36.40157 | -5.22023 |
| SB63832 | 01/08/2019 | 12:00:11 | 36.15265 | -5.83342 |
| SB63832 | 05/08/2019 | 12:00:11 | 36.4202 | -5.40688 |
| SB63832 | 09/08/2019 | 12:00:09 | 36.5031 | -5.19394 |
| SB63832 | 13/08/2019 | 12:00:10 | 36.2542 | -5.88345 |
| SB63832 | 17/08/2019 | 12:00:07 | 36.12148 | -5.81868 |
| SB63832 | 21/08/2019 | 12:00:11 | 36.75694 | -6.44415 |
| SB63832 | 25/08/2019 | 12:00:11 | 37.01315 | -5.74614 |
| SB63832 | 29/08/2019 | 12:00:08 | 35.19155 | -5.74387 |
| SB63832 | 02/09/2019 | 12:00:04 | 18.36456 | -15.1451 |
| SB63832 | 06/09/2019 | 12:00:04 | 16.55615 | -8.64913 |
| SB63832 | 10/09/2019 | 12:00:04 | 16.87798 | 2.07036 |
| SB63832 | 14/09/2019 | 12:00:04 | 15.7567 | 3.06937 |
| SB63832 | 18/09/2019 | 12:00:06 | 17.04448 | 4.15599 |
| SB63832 | 22/09/2019 | 12:00:04 | 15.92273 | 3.10677 |
| SB63832 | 26/09/2019 | 12:00:10 | 15.98745 | 0.79892 |
| SB63832 | 30/09/2019 | 12:00:04 | 15.55807 | 1.53607 |
| SB63832 | 04/10/2019 | 12:00:11 | 15.92396 | 2.91432 |
| SB63832 | 08/10/2019 | 12:00:06 | 17.02866 | 0.94123 |
| SB63832 | 12/10/2019 | 12:00:06 | 19.03656 | -7.87201 |
| SB63832 | 16/10/2019 | 12:00:06 | 15.93792 | -2.31836 |
| SB63832 | 20/10/2019 | 12:00:06 | 16.46747 | -4.54023 |
| SB63832 | 24/10/2019 | 12:00:05 | 18.2834 | -2.32657 |
| SB63832 | 28/10/2019 | 12:00:05 | 16.91542 | 5.5434 |
| SB63832 | 01/11/2019 | 12:00:06 | 15.75977 | 2.71145 |
| SB63832 | 05/11/2019 | 12:00:06 | 17.35465 | -0.23829 |
| SB63832 | 09/11/2019 | 12:00:04 | 16.679 | 4.63096 |
| SB63832 | 13/11/2019 | 12:00:04 | 11.38558 | 0.46148 |
| SB63832 | 17/11/2019 | 12:00:04 | 9.37762 | 4.09987 |
| SB63832 | 21/11/2019 | 12:00:04 | 11.40706 | 5.83806 |
| SB63832 | 25/11/2019 | 12:00:04 | 11.65944 | 6.02838 |
| SB63832 | 29/11/2019 | 12:00:04 | 9.32276 | 6.10558 |
| SB63832 | 03/12/2019 | 12:00:05 | 6.74543 | 5.74581 |
| SB63832 | 07/12/2019 | 12:00:05 | 6.62009 | 5.54265 |
| SB63832 | 11/12/2019 | 12:00:04 | 6.88055 | 5.66664 |
| SB63832 | 15/12/2019 | 12:00:04 | 6.87148 | 5.26432 |
| SB63832 | 19/12/2019 | 12:00:06 | 7.09686 | 4.74791 |
| SB63832 | 23/12/2019 | 12:00:05 | 5.86951 | 5.94384 |
| SB63832 | 27/12/2019 | 12:00:04 | 4.72798 | 9.25505 |
| SB63832 | 31/12/2019 | 12:00:05 | 5.31154 | 9.39072 |
| SB63832 | 04/01/2020 | 12:00:04 | 4.6715 | 9.18892 |
| SB63832 | 08/01/2020 | 12:00:04 | 5.94738 | 9.90938 |
| SB63832 | 12/01/2020 | 12:00:08 | 6.36741 | 8.89954 |
| SB63832 | 16/01/2020 | 12:00:06 | 5.21289 | 8.64843 |
| SB63832 | 20/01/2020 | 12:00:06 | 6.28685 | 9.76349 |
| SB63832 | 24/01/2020 | 12:00:09 | 7.15731 | 9.58433 |
| SB63832 | 28/01/2020 | 12:00:04 | 6.78387 | 10.88153 |
| SB63832 | 01/02/2020 | 12:00:06 | 5.28453 | 10.67652 |
| SB63832 | 05/02/2020 | 12:00:07 | 5.93906 | 10.91673 |
| SB63832 | 09/02/2020 | 12:00:04 | 5.0766 | 10.32074 |
| SB63832 | 13/02/2020 | 12:00:05 | 4.60907 | 10.66462 |
| SB63832 | 17/02/2020 | 12:00:06 | 6.12351 | 10.95622 |
| SB63832 | 21/02/2020 | 12:00:07 | 3.95436 | 9.91435 |
| SB63832 | 25/02/2020 | 12:00:06 | 5.81304 | 9.19738 |
| SB63832 | 29/02/2020 | 12:00:06 | 4.39888 | 9.3778 |
| SB63832 | 04/03/2020 | 12:00:11 | 6.08284 | 9.52952 |
| SB63832 | 08/03/2020 | 12:00:10 | 4.73816 | 9.6176 |
| SB63832 | 12/03/2020 | 12:00:07 | 6.761 | 10.01492 |
| SB63832 | 16/03/2020 | 12:00:04 | 5.49475 | 8.62643 |
| SB63832 | 20/03/2020 | 12:00:07 | 6.27346 | 9.62901 |
| SB63832 | 24/03/2020 | 12:00:04 | 6.68784 | 10.19826 |
| SB63832 | 28/03/2020 | 12:00:04 | 6.74897 | 10.56349 |
| SB63832 | 01/04/2020 | 12:00:06 | 7.24216 | 11.36215 |
| SB63832 | 05/04/2020 | 12:00:10 | 20.17538 | 2.33656 |
| SB63832 | 09/04/2020 | 12:00:09 | 8.37771 | -3.53865 |
| SB63832 | 13/04/2020 | 12:00:04 | 15.45781 | -3.75367 |
| SB63832 | 17/04/2020 | 12:00:06 | 36.08401 | -5.6149 |
|  |  |  |  |  |
| SB63835 | 17/08/2019 | 12:00:04 | 25.32901 | -11.6151 |
| SB63835 | 21/08/2019 | 12:00:11 | 14.9997 | -13.0033 |
| SB63835 | 25/08/2019 | 12:00:07 | 18.40321 | -8.68273 |
| SB63835 | 29/08/2019 | 12:00:08 | 18.08042 | -8.36518 |
| SB63835 | 02/09/2019 | 12:00:07 | 17.17035 | -6.85607 |
| SB63835 | 06/09/2019 | 12:00:07 | 16.82124 | -3.79448 |
| SB63835 | 10/09/2019 | 12:00:10 | 16.7757 | -3.83004 |
| SB63835 | 14/09/2019 | 12:00:04 | 16.81011 | -3.41633 |
| SB63835 | 18/09/2019 | 12:00:06 | 17.9867 | -5.73605 |
| SB63835 | 22/09/2019 | 12:00:07 | 17.02107 | -5.63554 |
| SB63835 | 26/09/2019 | 12:00:07 | 16.91593 | -5.13216 |
| SB63835 | 30/09/2019 | 12:00:04 | 16.31542 | -4.2031 |
| SB63835 | 04/10/2019 | 12:00:05 | 16.34957 | -4.59705 |
| SB63835 | 08/10/2019 | 12:00:06 | 16.11552 | -4.66365 |
| SB63835 | 12/10/2019 | 12:00:06 | 16.61976 | -9.61266 |
| SB63835 | 16/10/2019 | 12:00:04 | 16.32459 | -8.80772 |
| SB63835 | 20/10/2019 | 12:00:05 | 15.96271 | -9.42388 |
| SB63835 | 24/10/2019 | 12:00:04 | 14.90563 | -10.9135 |
| SB63835 | 28/10/2019 | 12:00:07 | 13.08611 | -17.1702 |
| SB63835 | 01/11/2019 | 12:00:07 | 13.16499 | -15.0389 |
| SB63835 | 05/11/2019 | 12:00:04 | 14.34371 | -12.6351 |
| SB63835 | 09/11/2019 | 12:00:05 | 14.16033 | -12.8402 |
| SB63835 | 13/11/2019 | 12:00:05 | 14.666 | -12.4653 |
| SB63835 | 17/11/2019 | 12:00:04 | 14.28592 | -12.7663 |
| SB63835 | 21/11/2019 | 12:00:04 | 10.88693 | -14.4405 |
| SB63835 | 25/11/2019 | 12:00:05 | 12.30551 | -15.9622 |
| SB63835 | 29/11/2019 | 12:00:05 | 11.52856 | -14.6617 |
| SB63835 | 03/12/2019 | 12:00:11 | 12.44911 | -15.7805 |
| SB63835 | 07/12/2019 | 12:00:11 | 7.20345 | -11.7533 |
| SB63835 | 11/12/2019 | 12:00:09 | 8.2225 | -12.1606 |
| SB63835 | 15/12/2019 | 12:00:06 | 7.99318 | -12.459 |
| SB63835 | 19/12/2019 | 12:00:11 | 7.98389 | -11.6167 |
| SB63835 | 23/12/2019 | 12:00:04 | 7.99504 | -11.627 |
| SB63835 | 27/12/2019 | 12:00:05 | 7.89711 | -12.4359 |
| SB63835 | 31/12/2019 | 12:00:04 | 8.09487 | -12.0601 |
| SB63835 | 04/01/2020 | 12:00:09 | 8.76053 | -12.9093 |
| SB63835 | 08/01/2020 | 12:00:06 | 7.2534 | -11.263 |
| SB63835 | 12/01/2020 | 12:00:11 | 10.84412 | -11.3236 |
| SB63835 | 16/01/2020 | 12:00:07 | 8.23113 | -12.347 |
| SB63835 | 20/01/2020 | 12:00:07 | 7.96394 | -12.465 |
| SB63835 | 24/01/2020 | 12:00:10 | 6.71202 | -10.8613 |
