# Supplementary material for: Birds with multiple homes. The annual cycle of the pallid swift (Apus pallidus brehmorum)
Source: PLoS One. 2021 Nov 30;16(11):e0259656. doi: 10.1371/journal.pone.0259656 (PMC8631615; doi:10.1371/journal.pone.0259656)
Supplement: S2 Table — Data show that the birds remained airborne throughout, including at night. (DOCX) [file pone.0259656.s002.docx]

S2 Table. Data of two Pallid Swifts during the 2020-21 non-breeding season showing that the birds remained airborne throughout, including at night.

| **Ring No.** | **Tag** | **Date** | **Time** | **Latitude** | **Longitude** | **Altitude** |
| --- | --- | --- | --- | --- | --- | --- |
| SB63853 | 49556 | 18/08/2020 | 12:00:09 | 36.4328 | -5.74792 | 1226.45 |
| SB63853 | 49556 | 26/08/2020 | 00:00:06 | NOT ENOUGH SATS | | |
| SB63853 | 49556 | 26/08/2020 | 12:00:11 | 36.47413 | -6.02856 | 156.33 |
| SB63853 | 49556 | 03/09/2020 | 00:00:04 | 34.67865 | -7.1876 | 81.42 |
| SB63853 | 49556 | 03/09/2020 | 12:00:11 | 32.98121 | -8.60533 | 389.21 |
| SB63853 | 49556 | 11/09/2020 | 00:00:05 | NOT ENOUGH SATS | | |
| SB63853 | 49556 | 11/09/2020 | 12:00:06 | 18.25868 | -12.8571 | 823.4 |
| SB63853 | 49556 | 19/09/2020 | 00:00:04 | 18.16589 | -14.491 | 286.36 |
| SB63853 | 49556 | 19/09/2020 | 12:00:04 | 18.23207 | -13.4099 | 1678.56 |
| SB63853 | 49556 | 27/09/2020 | 00:00:04 | 17.25871 | -8.10366 | 686.98 |
| SB63853 | 49556 | 27/09/2020 | 12:00:08 | 17.99826 | -6.04314 | 1621.3 |
| SB63853 | 49556 | 05/10/2020 | 00:00:04 | 16.69478 | -9.56608 | 1025.69 |
| SB63853 | 49556 | 05/10/2020 | 12:00:07 | 17.44826 | -9.06602 | 733.22 |
| SB63853 | 49556 | 13/10/2020 | 00:00:05 | 18.53679 | -16.2807 | 382.42 |
| SB63853 | 49556 | 13/10/2020 | 12:00:08 | 17.4904 | -16.3393 | -38.74 |
| SB63853 | 49556 | 21/10/2020 | 00:00:04 | 16.10381 | -16.1732 | 249.17 |
| SB63853 | 49556 | 21/10/2020 | 12:00:06 | 15.44631 | -14.9275 | 875.52 |
| SB63853 | 49556 | 29/10/2020 | 00:00:04 | 16.88972 | -16.3717 | 447.31 |
| SB63853 | 49556 | 29/10/2020 | 12:00:04 | 15.84102 | -16.1525 | 444.37 |
| SB63853 | 49556 | 06/11/2020 | 00:00:09 | 18.76344 | -13.6344 | 1007.74 |
| SB63853 | 49556 | 06/11/2020 | 12:00:08 | 17.66018 | -13.1188 | 340.45 |
| SB63853 | 49556 | 14/11/2020 | 00:00:04 | 13.74998 | -14.9371 | 446.43 |
| SB63853 | 49556 | 14/11/2020 | 12:00:07 | 13.34751 | -15.1871 | 529.82 |
| SB63853 | 49556 | 22/11/2020 | 00:00:04 | 13.87888 | -11.9146 | 565.36 |
| SB63853 | 49556 | 22/11/2020 | 12:00:07 | 14.89743 | -11.6125 | 1249.73 |
| SB63853 | 49556 | 30/11/2020 | 00:00:03 | 13.78478 | -12.5073 | 1636.33 |
| SB63853 | 49556 | 30/11/2020 | 12:00:04 | 13.63459 | -11.9928 | 780.54 |
| SB63853 | 49556 | 08/12/2020 | 00:00:04 | 12.59212 | -13.4932 | 610.12 |
| SB63853 | 49556 | 08/12/2020 | 12:00:07 | 13.38295 | -13.1641 | 767.81 |
| SB63853 | 49556 | 16/12/2020 | 00:00:04 | 15.01046 | -12.2084 | 997.81 |
| SB63853 | 49556 | 16/12/2020 | 12:00:06 | 15.66097 | -11.4121 | 352.74 |
| SB63853 | 49556 | 24/12/2020 | 00:00:06 | 12.40908 | -12.8375 | 1250 |
| SB63853 | 49556 | 24/12/2020 | 12:00:11 | 11.88174 | -11.5241 | 2394.15 |
| SB63853 | 49556 | 01/01/2021 | 00:00:04 | 6.36083 | -7.84099 | 1519.54 |
| SB63853 | 49556 | 01/01/2021 | 12:00:07 | 7.60584 | -8.0745 | 1272.01 |
| SB63853 | 49556 | 09/01/2021 | 00:00:04 | 6.87796 | -8.46953 | 735.75 |
| SB63853 | 49556 | 09/01/2021 | 12:00:08 | 7.37094 | -7.75917 | 1093.13 |
| SB63853 | 49556 | 17/01/2021 | 00:00:04 | 6.74815 | -7.77283 | 817.85 |
| SB63853 | 49556 | 17/01/2021 | 12:00:06 | 5.96046 | -7.63031 | 411.36 |
| SB63853 | 49556 | 25/01/2021 | 00:00:04 | 8.60715 | -7.85466 | 1043.72 |
| SB63853 | 49556 | 25/01/2021 | 12:00:11 | 8.99759 | -7.49107 | 648.88 |
| SB63853 | 49556 | 02/02/2021 | 00:00:05 | 6.82344 | -7.85767 | 1082.91 |
| SB63853 | 49556 | 02/02/2021 | 12:00:10 | 8.55151 | -6.95745 | 494.54 |
| SB63853 | 49556 | 10/02/2021 | 00:00:04 | 7.19696 | -6.16597 | 439.2 |
| SB63853 | 49556 | 10/02/2021 | 12:00:11 | 6.27765 | -7.17929 | 572.64 |
| SB63853 | 49556 | 18/02/2021 | 00:00:05 | 7.82416 | -6.74991 | 649.42 |
| SB63853 | 49556 | 18/02/2021 | 12:00:08 | 6.87847 | -6.27751 | 1044.53 |
| SB63853 | 49556 | 26/02/2021 | 00:00:04 | 7.65187 | -7.16943 | 1399.72 |
| SB63853 | 49556 | 26/02/2021 | 12:00:04 | 7.78898 | -6.85524 | 875.2 |
| SB63853 | 49556 | 06/03/2021 | 00:00:04 | 8.01666 | -7.29433 | 1065.11 |
| SB63853 | 49556 | 06/03/2021 | 12:00:07 | 8.62657 | -7.72561 | 735.23 |
| SB63853 | 49556 | 14/03/2021 | 00:00:04 | 19.30262 | -11.7662 | 1300.27 |
|  |  |  |  |  |  |  |
| SB63835 | 50198 | 08/08/2020 | 00:00:11 | 36.58321 | -5.85957 | 616.84 |
| SB63835 | 50198 | 12/08/2020 | 00:00:05 | 33.7549 | -5.02714 | 1438.42 |
| SB63835 | 50198 | 16/08/2020 | 00:00:06 | 36.47422 | -5.572 | 727.8 |
| SB63835 | 50198 | 20/08/2020 | 00:00:05 | 36.11342 | -5.42185 | 1145.86 |
| SB63835 | 50198 | 24/08/2020 | 00:00:06 | 19.02115 | -14.8657 | 192.87 |
| SB63835 | 50198 | 28/08/2020 | 00:00:04 | 17.98001 | -10.6174 | 572.17 |
| SB63835 | 50198 | 01/09/2020 | 00:00:05 | 17.16619 | -3.80814 | 715.3 |
| SB63835 | 50198 | 05/09/2020 | 00:00:06 | 17.944 | -5.72602 | 648.31 |
| SB63835 | 50198 | 09/09/2020 | 00:00:06 | 17.49169 | -4.36064 | 1119.13 |
| SB63835 | 50198 | 13/09/2020 | 00:00:06 | 17.34008 | -4.6137 | 388.66 |
| SB63835 | 50198 | 17/09/2020 | 00:00:06 | 16.84923 | -3.69373 | 701.39 |
| SB63835 | 50198 | 21/09/2020 | 00:00:06 | 16.40743 | -4.43715 | 2983.32 |
| SB63835 | 50198 | 25/09/2020 | 00:00:07 | 16.80835 | -3.64066 | 806.98 |
| SB63835 | 50198 | 29/09/2020 | 00:00:05 | 17.31313 | -4.88642 | 1395.42 |
| SB63835 | 50198 | 03/10/2020 | 00:00:04 | 17.08621 | -4.09668 | 892.37 |
| SB63835 | 50198 | 07/10/2020 | 00:00:06 | 16.24993 | -5.82363 | 1032.74 |
| SB63835 | 50198 | 11/10/2020 | 00:00:06 | 17.21684 | -8.14823 | 595.03 |
| SB63835 | 50198 | 15/10/2020 | 00:00:06 | 16.40157 | -9.52754 | 455.94 |
| SB63835 | 50198 | 19/10/2020 | 00:00:08 | 15.73661 | -9.83214 | 405.6 |
| SB63835 | 50198 | 23/10/2020 | 00:00:07 | 16.00647 | -8.90808 | 722.56 |
| SB63835 | 50198 | 27/10/2020 | 00:00:06 | 16.24597 | -14.3147 | 779.54 |
| SB63835 | 50198 | 31/10/2020 | 00:00:06 | 12.32433 | -16.8995 | -49.52 |
| SB63835 | 50198 | 04/11/2020 | 00:00:04 | 14.72379 | -15.3537 | 1068.41 |
| SB63835 | 50198 | 08/11/2020 | 00:00:09 | 13.34402 | -14.9726 | 1259.58 |
| SB63835 | 50198 | 12/11/2020 | 00:00:05 | 13.52884 | -16.6173 | 314.99 |
| SB63835 | 50198 | 16/11/2020 | 00:00:05 | 13.99308 | -14.6427 | 182.51 |
| SB63835 | 50198 | 20/11/2020 | 00:00:04 | 13.69174 | -13.3204 | 271.79 |
| SB63835 | 50198 | 24/11/2020 | 00:00:04 | 13.69593 | -14.0838 | 1120.4 |
| SB63835 | 50198 | 28/11/2020 | 00:00:04 | 13.12795 | -13.6006 | 1254.6 |
| SB63835 | 50198 | 02/12/2020 | 00:00:04 | 13.71522 | -13.7027 | 1444.74 |
| SB63835 | 50198 | 06/12/2020 | 00:00:04 | 11.58224 | -14.5142 | 980.93 |
| SB63835 | 50198 | 10/12/2020 | 00:00:04 | 10.82599 | -14.986 | 765.53 |
| SB63835 | 50198 | 14/12/2020 | 00:00:06 | 10.34737 | -14.6368 | 114.87 |
| SB63835 | 50198 | 18/12/2020 | 00:00:05 | 9.40681 | -14.6799 | 110.43 |
| SB63835 | 50198 | 22/12/2020 | 00:00:04 | 6.93214 | -11.704 | 18.99 |
| SB63835 | 50198 | 26/12/2020 | 00:00:04 | 7.47647 | -12.7807 | 97.26 |
| SB63835 | 50198 | 30/12/2020 | 00:00:04 | 8.18006 | -13.2551 | -207.24 |
| SB63835 | 50198 | 03/01/2021 | 00:00:04 | 8.86557 | -12.2847 | 2143.61 |
| SB63835 | 50198 | 07/01/2021 | 00:00:05 | 7.15303 | -10.9031 | 420.6 |
| SB63835 | 50198 | 11/01/2021 | 00:00:04 | 8.6054 | -11.1124 | 767 |
| SB63835 | 50198 | 15/01/2021 | 00:00:05 | 7.82532 | -11.2249 | 588.86 |
| SB63835 | 50198 | 19/01/2021 | 00:00:04 | 8.78509 | -13.3689 | 55.87 |
| SB63835 | 50198 | 23/01/2021 | 00:00:07 | 7.69577 | -12.8322 | 1199.29 |
| SB63835 | 50198 | 27/01/2021 | 00:00:05 | 7.54119 | -11.5409 | 1919.28 |
| SB63835 | 50198 | 31/01/2021 | 00:00:04 | 7.99788 | -11.1207 | 1218.91 |
| SB63835 | 50198 | 04/02/2021 | 00:00:05 | 7.59428 | -7.71313 | 1159.48 |
| SB63835 | 50198 | 08/02/2021 | 00:00:06 | 7.37125 | -8.4541 | 614.89 |
| SB63835 | 50198 | 12/02/2021 | 00:00:04 | 8.33882 | -10.7365 | 559.98 |
| SB63835 | 50198 | 16/02/2021 | 00:00:06 | 7.78781 | -11.4527 | 309.53 |
| SB63835 | 50198 | 20/02/2021 | 00:00:07 | 7.99912 | -10.9509 | 853.76 |
| SB63835 | 50198 | 24/02/2021 | 00:00:07 | 7.73809 | -12.7197 | 1263.9 |
| SB63835 | 50198 | 28/02/2021 | 00:00:05 | 5.6179 | -7.37383 | 1736.96 |
| SB63835 | 50198 | 04/03/2021 | 00:00:07 | 7.36254 | -7.99304 | 2126.18 |
| SB63835 | 50198 | 08/03/2021 | 00:00:06 | 8.46359 | -8.87858 | 1206.24 |
| SB63835 | 50198 | 12/03/2021 | 00:00:04 | 8.74443 | -9.26695 | 1160.28 |
| SB63835 | 50198 | 16/03/2021 | 00:00:04 | 8.15067 | -10.335 | 590.07 |
| SB63835 | 50198 | 20/03/2021 | 00:00:06 | 9.03474 | -6.48093 | 833.63 |
| SB63835 | 50198 | 24/03/2021 | 00:00:07 | 9.32908 | -8.3287 | 1858.48 |
| SB63835 | 50198 | 28/03/2021 | 00:00:04 | 6.99803 | -11.1367 | 1630.89 |
| SB63835 | 50198 | 01/04/2021 | 00:00:04 | 9.67299 | -10.6348 | 2062.36 |
| SB63835 | 50198 | 05/04/2021 | 00:00:05 | NOT ENOUGH SATS | | |
| SB63835 | 50198 | 09/04/2021 | 00:00:06 | 36.10586 | -5.62539 | 323.66 |
| SB63835 | 50198 | 13/04/2021 | 00:00:06 | 36.10436 | -5.6127 | 572.13 |
